# Structures to the people!

**DOI:** 10.7554/eLife.09249

**Published:** 2015-07-08

**Authors:** Nathan J Baird, Sebastian A Leidel

**Affiliations:** Department of Chemistry and Biochemistry, University of the Sciences, Philadelphia, United States; RNA Biology Laboratory, Max Planck Institute for Molecular Biomedicine, Muenster, Germany and the Cells-in-Motion Cluster of Excellence, Faculty of Medicine, University of Muenster, Muenster, Germanysebastian.leidel@mpi-muenster.mpg.de

**Keywords:** non-coding RNA, riboswitches, ribozymes, structure prediction, next-generation sequencing, high-throughput, none

## Abstract

A combination of 3D modeling and high-throughput sequencing may offer a faster way to determine the three-dimensional structures of RNA molecules.

**Related research article** Cheng CY, Chou FC, Kladwang W, Tian S, Cordero P, Das R. 2015. Consistent global structures of complex RNA states through multidimensional chemical mapping. *eLife* **4**:e07600. doi: 10.7554/eLife.07600

**Image** Multidimensional chemical mapping predicts the structures of RNAs based on interactions between nucleotides

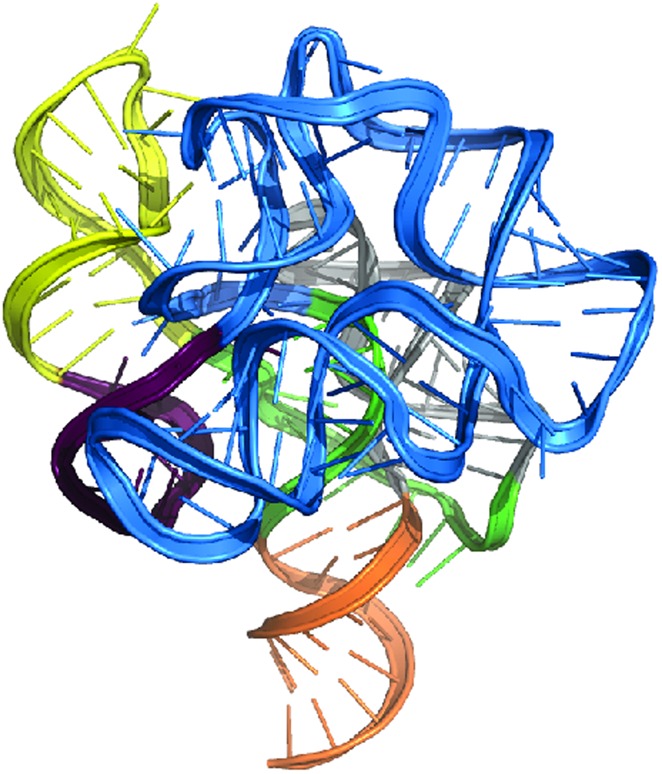

The structures of molecules often hold the key to understanding their roles in cells. Thus, when Watson and Crick proposed the double-helix structure for DNA, they immediately speculated on how DNA may replicate. Unfortunately, working out the structures of RNA molecules is challenging, and the techniques needed to do so—X-ray crystallography and NMR—are only available at a small number of locations worldwide. This has prevented many small laboratories from embracing structural work.

Instead, some researchers have started to predict the three-dimensional structures of RNA molecules based on the sequence of nucleotides in the molecule, and there is even an ‘RNA Puzzles’ competition to compare the performance of prediction algorithms (Cruz et al., 2012; Miao et al., 2015). Moreover, the discovery of large numbers of non-coding RNAs in recent years has substantially increased the need for methods that can determine the structure of RNA molecules quickly and reliably. Now, in *eLife*, Rhiju Das and co-workers—including Clarence Yu Cheng as first author—describe a new approach that promises to simplify the determination of RNA structures to nanometer resolution (Cheng et al., 2015).

This approach, which is called multidimensional chemical mapping, can be likened to the power of social networking. In social networks, centralized ‘hot spots’ of communication can be identified, based on how well connected they are, and in some cases these hot spots can influence, or constrain, the activity of large numbers of other individuals in the network. Similarly, the approach developed by Cheng et al., who are based at Stanford University, identifies the three-dimensional networks of interactions between nucleotides within a given RNA molecule to guide the predictions of its structure.

Interactions between nucleotides in most RNA molecules lead to secondary structures called helices, which include loops, bulged nucleotides and multi-helical junctions. In turn, these secondary structure elements influence how the molecule folds into its final three-dimensional (or tertiary) structure. Previously, Das and others have combined protocols that predict the secondary structure of RNA with three-dimensional modelling algorithms (Kladwang et al., 2011; Cruz et al., 2012; Miao et al., 2015) to generate models of the tertiary structures of RNAs. However, the relative orientations of the secondary structure elements could not be precisely defined due to a lack of experimental data on the three-dimensional structure.

Several experimental approaches can fill this gap, but many require specialized reagents or equipment, which limits their use. Just as the growth of social media depended on developments in technological infrastructure, the current progress in multidimensional chemical mapping has been made possible by the widespread availability of instruments for deep sequencing DNA. In a wry twist of fate, sequencing has become a key tool for defining structure.

The idea to use sequencing to determine RNA structures is not new (see review by Ge and Zhang, 2015), but to date the focus has been on predicting secondary structure. Recently, however, Kevin Weeks and co-workers went a step further and used correlated data between nucleotides to predict tertiary structure, although they only reported on the interactions between two nucleotides, cytosine and adenosine (Homan et al., 2014). Cheng et al. go even further by considering potentially all of the interactions between all four of the nucleotides.

Multidimensional chemical mapping combines a technique called MOHCA (short for multiplexed •OH cleavage analysis) with deep sequencing and algorithms that predict the RNA secondary and tertiary structure (Figure 1). MOHCA uses RNA molecules that contain chemically modified nucleotides, usually one per molecule, at random positions. When the RNA molecules are treated with certain chemicals, highly reactive molecules called hydroxyl radicals are produced, and these cleave the RNA molecule near the site of the modified nucleotide. This effectively marks the positions of other nucleotides that are found near the modified nucleotide in the three-dimensional structure of the RNA molecule.

**Figure 1.**
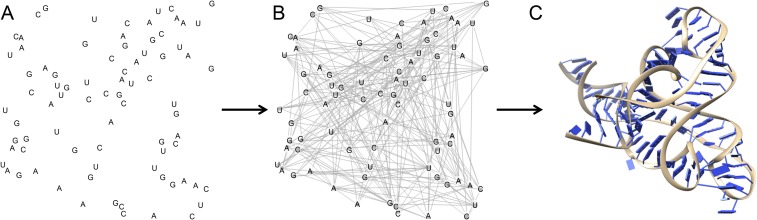
The three dimensional structure of an RNA molecule can be predicted by combining MOHCA, deep sequencing and algorithms that predict secondary and tertiary structures in the RNA. (**A**) In MOHCA, copies of the RNA of interest that contain modified nucleotides—on average one per molecule—are made. These modified nucleotides produce hydroxyl groups that cleave the RNA and damage other nucleotides near to the modified nucleotide. A reverse transcriptase enzyme is used to generate DNA copies of the RNA molecules. This enzyme stops copying each RNA molecule at the point where it is cleaved or damaged. Therefore, sequencing these DNA fragments reveals the positions of nucleotides that are close to the modified nucleotide in the three-dimensional structure. This information is used to make a network of the interactions between all the nucleotides in the RNA (**B**). This network is then combined with algorithms that predict the secondary and tertiary structures to produce a single three-dimensional model of the tertiary structure (**C**). Image prepared by Nathan Baird using CodePen (codepen.io/blendmaster/full/uqibt) and structure 2YIE from the Protein Data Bank.

Previously, gel electrophoresis has been used to show where the RNA molecule is cleaved during MOHCA, but combining MOHCA with deep sequencing provides much more detailed information and significantly increases throughput. Cheng et al. constructed maps showing the interactions between the nucleotides in the molecule and combined these with algorithms that predict secondary and tertiary structures to generate a well-defined three-dimensional model of the RNA. To demonstrate the accuracy and utility of this new approach, Cheng et al. applied it to five RNAs of known structure, and to one RNA whose structure had not been released at the time. Multidimensional chemical mapping successfully predicted the three-dimensional structures of all six RNA molecules to within nanometer resolution.

However, RNA often interacts with cofactors, which remain largely undefined or otherwise make structure determination more challenging. Multidimensional chemical mapping works in solution and can help define structures in the absence of these cofactors. To test this, Cheng et al. used multidimensional chemical mapping to predict the structures of several RNAs without their cellular cofactors. Their prediction for the structure of the internal ribosomal entry site in human Hox messenger RNA will aid efforts to determine the structure of this mRNA in complex with the ribosome and other partner molecules using techniques such as cryo-electron microscopy. Cheng et al. also modeled ligand-free conformations of several riboswitches, which may guide the development of drugs that stabilize non-functional RNA conformations.

But what challenges are still ahead of us? Currently, the size of the RNA limits the analysis. Refining the modeling algorithm and the way the samples are prepared for sequencing may help to improve the predictions. A more general concern is the presence of naturally occurring modifications to RNA molecules inside cells. In highly modified structures—like transfer RNAs—these chemical groups influence the folding of the molecule. Thus, when solving the structure of an unmodified RNA molecule produced in an artificial system, an important piece in the puzzle is missing. Even when they are present, how these chemical modifications affect the cleavage of RNA by the hydroxyl radicals still needs to be assessed.

The field of structural biology is undergoing dramatic changes as improvements in technology—such as the development of free-electron lasers and improved detectors for electron microscopy—are making it possible to solve structures at atomic resolution, almost in a high-throughput manner. Will the combination of three-dimensional modeling and sequencing become a similar game changer in the field of RNA structures, being used and adapted by large numbers of researchers? Multidimensional chemical mapping clearly has the potential to transform structure determination by putting it into the hands of researchers who have experience in molecular biology and access to deep sequencing.

Today, this is almost everybody: structures to the people!
